# Nucleus reticularis tegmenti pontis: a bridge between the basal ganglia and cerebellum for movement control

**DOI:** 10.1007/s00221-023-06574-0

**Published:** 2023-03-31

**Authors:** Alan R. Gibson, Kris M. Horn, Milton Pong

**Affiliations:** 1 Barrow Neurological Institute, St. Joseph’s Hospital and Medical Center, Phoenix, AZ 85013 USA; 2 Present Address: 3417 E. Mission Ln, Phoenix, AZ 85028 USA; 3 Present Address: Chamberlain College of Nursing, 1036 E Baylor Ln, Gilbert, AZ 85296 USA; 4 Present Address: School of Osteopathic Medicine, Arizona, A. T. Still University, 5850 E. Still Circle, Mesa, AZ 85206 USA

**Keywords:** Cerebellum, Basal ganglia, Nucleus reticularis tegmenti pontis (NRTP), Parkinon’s Disease, Limb movement, Cat

## Abstract

Neural processing in the basal ganglia is critical for normal movement. Diseases of the basal ganglia, such as Parkinson’s disease, produce a variety of movement disorders including akinesia and bradykinesia. Many believe that the basal ganglia influence movement via thalamic projections to motor areas of the cerebral cortex and through projections to the cerebellum, which also projects to the motor cortex via the thalamus. However, lesions that interrupt these thalamic pathways to the cortex have little effect on many movements, including limb movements. Yet, limb movements are severely impaired by basal ganglia disease or damage to the cerebellum. We can explain this impairment as well as the mild effects of thalamic lesions if basal ganglia and cerebellar output reach brainstem motor regions without passing through the thalamus. In this report, we describe several brainstem pathways that connect basal ganglia output to the cerebellum via nucleus reticularis tegmenti pontis (NRTP). Additionally, we propose that widespread afferent and efferent connections of NRTP with the cerebellum could integrate processing across cerebellar regions. The basal ganglia could then alter movements via descending projections of the cerebellum. Pathways through NRTP are important for the control of normal movement and may underlie deficits associated with basal ganglia disease.

## Introduction

Parkinson’s disease (PD), as well as other diseases of the basal ganglia, are characterized by disturbances in movement control. It is generally believed that movements are altered via basal ganglia effects on the motor cortex. The basal ganglia do not directly project to the motor cortex, although they may influence the motor cortex via projections to the thalamus. Basal ganglia output largely targets nuclei in the thalamus that do not project to the motor cortex (Ilinsky and Kultas-Ilinsky 1984; Sakai et al. 1996), although there is some overlap with regions of the thalamus that project to the motor cortex (Sakai et al. 1996; Lanciego et al. 2012; Caligiore et al. 2017). These overlap regions may provide a path for modifying neural processing in the motor cortex. More recently, it has been demonstrated that basal ganglia output reaches the cerebellum (Bostan and Strick 2010, 2018). A pathway to the cerebellum could also allow basal ganglia output to modify the motor cortex since the output from the cerebellum targets thalamic nuclei that project to the motor cortex (Ilinsky and Kultas-Ilinsky 1984; Sakai et al. 1996). Therefore, basal ganglia output could modify motor cortex processing via multiple pathways projecting to the motor cortex via the thalamus.

However, there is a problem with movement pathways involving thalamic nuclei: Lesions in the thalamus that interrupt projections to the motor cortex can relieve tremors associated with PD or cerebellar disease but have little effect on other motor symptoms such as akinesia and bradykinesia (Marsden and Obeso 1994; Duval et al. 2006). Additionally, animal experiments show that limb movements, which are severely disturbed by basal ganglia diseases or cerebellar damage, remain relatively normal after large thalamic lesions (Canavan et al. 1989; Fabre-Thorpe and Levesque 1991) or temporary inactivation of the thalamus (van Donkelaar et al. 2000). Therefore, basal ganglia output must be able to influence movement via pathways that do not pass through the thalamus. Many structures in the brainstem are known to be targets of basal ganglia output (Edley and Graybiel 1983; McElvain et al. 2021). We propose that basal ganglia output to brainstem regions can alter movement without returning to the motor cortex via the thalamus.

One brainstem region implicated in basal ganglia control of movement is the pedunculopontine nucleus (PPN) (Aziz et al. 1998; French and Muthusamy 2018). Mori et al. (2016) proposed a projection from the PPN, which receives input from GPi (Edley and Graybiel 1983), to the cerebellar nuclei, thereby allowing the basal ganglia to influence movement via cerebellar projections to the motor thalamus. In the rat, there are cholinergic projections from PPN to the cerebellar nuclei and cortex, these projections may be important for the control of the arousal state (Inglis and Winn 1995; Jaarsma et al. 1997).

**Fig. 1:**
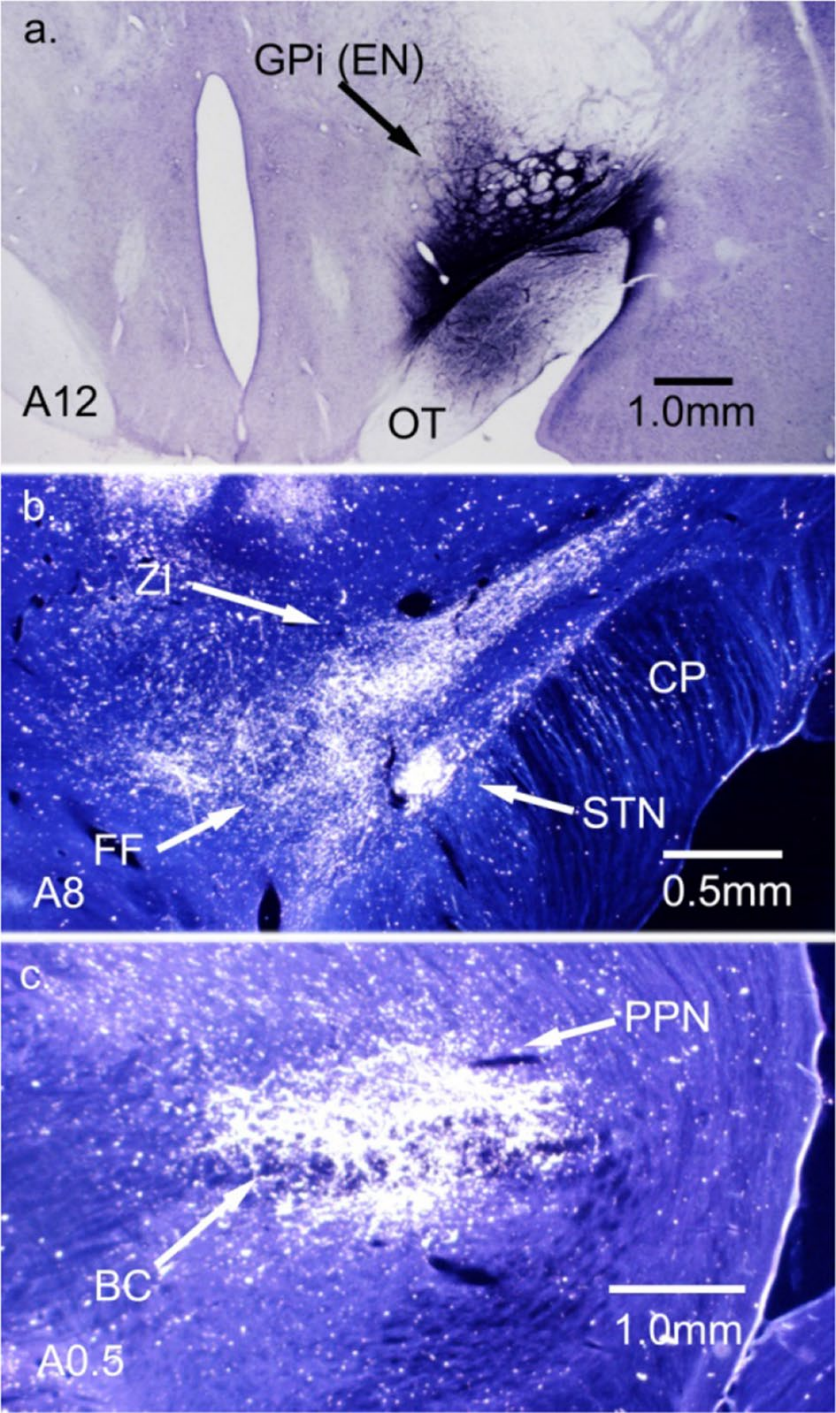
Two pathways in the cat that connect GPi output to the cerebellum. **a** GPi WGA-HRP injection (26nL, 1% WGA-HRP). **b** GPi terminations in ZI and FF. STN contains a large number of labeled cells. **c** GPi terminations in PPN surround and penetrate the brachium conjunctivum (BC). PPN and ZI/FF project to NRTP. Images in **b**, **c** Taken with darkfield polarized light. Frontal sections

PPN also projects to the nucleus reticularis tegmenti pontis (NRTP) (Edley and Graybiel 1983). NRTP has a very dense and widespread projection to cerebellar nuclei and cortex (Kawamura and Hashikawa 1981; Gerrits and Voogd 1986, 1987). Thus, the projection of the basal ganglia to PPN could provide a pathway to the cerebellar cortex and nuclei via NRTP. Figure 1 illustrates terminal labeling after a WGA-HRP injection into the cat entopeduncular nucleus (EN, feline equivalent to GPi), which is a major source of output from the basal ganglia. In addition to the PPN projection, NRTP receives input from several midbrain nuclei that could relay basal ganglia output to the cerebellum (Giolli et al. 2001).

NRTP has been studied largely in relation to eye movements, and activity in NRTP can have profound effects on eye movement control (Hess et al. 1989; Suzuki et al. 1999; Kaneko and Fuchs 2006). In addition to its projection to cerebellar regions involved in eye movement, NRTP projects to large regions of the cerebellum involved in movements of the entire body (Kawamura and Hashikawa 1981; Gerrits and Voogd 1986, 1987). In this report, we confirm that subthalamic, midbrain and pontine regions receive input from the basal ganglia and project to the cerebellum via NRTP. To do that, we identified inputs to NRTP by tracing retrogradely labeled cells after WGA-HRP injections into NRTP. Additional WGA-HRP injections were then made in those areas to confirm input to NRTP. and to identify pathways that might allow basal ganglia output to reach the injected areas. The results demonstrate that several midbrain and pontine regions could enable basal ganglia output to influence NRTP.

### Abbreviations

Depending upon the species, many of the structures cited in this report are labeled in other reports with different abbreviations. The following is a list of our abbreviations with common abbreviations for homologous structures used in other reports. Our use of abbreviations largely follows the nomenclature used by Berman (1968) and Berman and Jones (1982):

BC: Brachium Conjunctivum (Superior Cerebellar Peduncle).

BG: Basal Ganglia.

CP: Cerebral Peduncle.

GL: Granular Layer of Cerebellar Cortex.

GPi: Globus Pallidus pars internal (entopeduncular nucleus, EN).

FF: Fields of Forel (FF/ZI, with adjacent Zona Incerta).

FTC: Central Tegmental Field of the Midbrain.

FTP: Paralemniscal Tegmental Field.

HA: Hypothalamic Area.

IC: Inferior Colliculus.

IP: Cerebellar Posterior Interpositus Nucleus (NIP, IPP, INP).

IA: Cerebellar Anterior Interpositus Nucleus (NIA, IPA, INA).

L: Cerebellar Lateral Nucleus (Dentate Nucleus, CBL).

LVN: Lateral Vestibular Nucleus (Deiter’s Nucleus).

M: Cerebellar Medial Nucleus (Fastigial Nucleus, CBM).

NRTP: Nucleus Reticularis Tegmenti Pontis (RTP, RtTg, RTp).

OT: Optic Tract.

PAG: Periaqueductal Gray.

PD: Parkinson’s Disease.

PML: Paramedian Lobule of the Cerebellum.

PN: Pontine Nuclei (Basal Pontine Nucleus, PG).

PTA: Anterior Pretectal Nucleus.

PPN: Pedunculopontine Nucleus (TRC, TPc, Tegmenti Pedunculopontis pars compacta).

RNm: Magnocellular Red Nucleus.

SC: Superior Colliculus.

SNr: Substantia Nigra pars reticularis.

STN: Subthalamic Nucleus.

TMB: Tetramethyl Benzidine.

WGA-HRP: Wheatgerm Agglutinin conjugated with Horseradish Peroxidase.

ZI: Zona Incerta (ZI/FF with adjacent Fields of Forel).

## Methods

All research in this report was approved by the Institutional Animal Care and Use Committee at the Barrow Neurological Institute and conducted in accordance with both the National Institutes of Health’s Principles of Laboratory Animal Care (86–23, revised 1985) and the American Physiological Society’s Guiding Principles in the Care and Use of Animals.

### Anatomical studies

The anatomical data presented in this report were collected from studies performed at the Barrow Neurological Institute. We chose to use cats (8 total) as subjects since the anatomical data complemented our studies of neural activity in brainstem regions in cats trained to reach and grasp a lever. Although connections to NRTP were not the original target of the cases, we carefully plotted all brainstem labeling using the microscopic examination. Additional advantages of using cats as subjects include a large number of published anatomical studies using cats, a relatively large brain allowing good isolation of injected structures, and consistent skull anatomy for accurate stereotaxic localization.

Anesthesia consisted of an initial intramuscular injection of ketamine hydrochloride (8 mg/kg) followed by intravenous (iv) doses (10 mg) of pentobarbital sodium injected into the cephalic vein. When deep anesthesia was obtained, the cats were mounted into a Kopf stereotaxic frame. After a craniotomy, recordings with tungsten electrodes were used to identify the desired injection sites.

For tracing, we used pressure injections of 1% horseradish peroxidase conjugated to wheat germ agglutinin (WGA–HRP, Sigma). As a neural tracer, low concentrations of WGA–HRP offers several advantages. Typically, passing fibers do not incorporate enough tracer to label the parent cell body, and WGA–HRP is a sensitive anterograde and retrograde tracer, which allows input–output connections of the same injection to be accurately determined and minimizes the required number of subjects. Additionally, WGA–HRP transports rapidly, which makes it practical to maintain anesthesia during transport to avoid a recovery period followed by additional surgery. To confirm major sources of input to NRTP, we made additional WGA-HRP injections into regions containing large numbers of retrogradely labeled cells, which allowed us to see terminal labeling in NRTP as well as sources of input to the injected area.

Injection pipettes were accurately cross-referenced with tungsten recording electrodes using an optical zero point. Injection volumes were monitored with a microscope using a calibrated reticle, and injection volumes were in the nanoliter (nL) range (amount noted for each case in the text). Accuracy of the final placement was confirmed by recording through the tracer solution with the injection pipettes. Perfusion consisted of a saline rinse followed by two liters of freshly made paraformaldehyde (3–4%, depending upon the case) and a series of 10, 20 and 30% sucrose/phosphate buffer solutions (0.1 M, pH 7.4). In some cases, the rinse solution was 9.25% sucrose at a pressure of 300 mmHg followed by fixative and sucrose solutions at 120 mmHg (Cragg 1980). Brains were frozen and sectioned at 50 μm in either the parasagittal or frontal planes. Sections were processed with a modified tetramethyl benzidine (TMB) reaction (Gibson et al. 1984) and lightly stained with thionin.

**Fig. 2:**
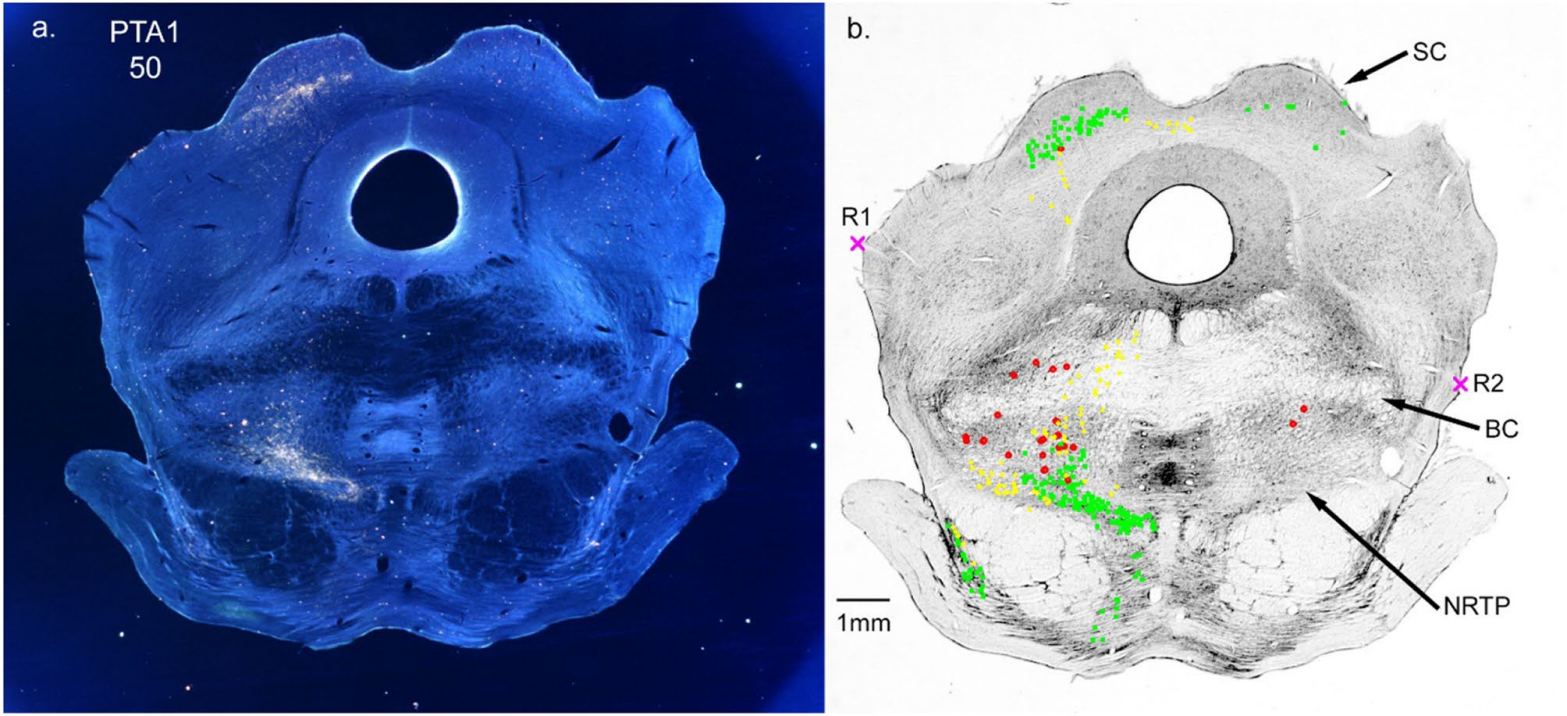
**a** Is a digital image of frontal section 50 from case PTA1 illuminated using darkfield polarized light. Patches of the terminal label are visible in the superior colliculus (SC) and in the nucleus reticularis tegmenti pontis (NRTP). **b** Is the same section imaged with brightfield illumination. TMB reaction product is not visible with brightfield illumination. The section was registered to the microscope stage position using reference points (R1x and R2x). Label was then marked under microscopic viewing using cross-polarized darkfield illumination. Retrogradely labeled cells were marked with red circles, anterogradely labeled fibers with yellow crosses and terminal regions with green x’s. The plotted image reveals more detail (i.e., labeled cells) than the dark field image

Except for densely labeled areas (such as injection sites), the largely transparent TMB reaction product was not visible under transmitted brightfield illumination. However, the crystalline TMB reaction product rotates polarized light and viewing under crossed polarized filters allowed us to see labeled structures as bright objects against a dark background. Figure 2a is a macro photo taken using cross-polarized illumination. The image is a frontal section through the midbrain following a WGA-HRP injection into the anterior pretectal nucleus (PTA, case PTA1, Fig. 10). Only dense areas of the terminal label can be discriminated in the macro image. Labeled cells, fibers and light terminal areas cannot be identified since the labeling appears the same for all structures, and low-power images do not have sufficient resolution to identify shapes of small structures. To resolve the different structures, it is necessary to examine the sections under high-powered (50-100x) magnification using cross-polarized illumination. To do that, the processed brain sections were digitized, and the digital images were cross-referenced to the microscope stage position by marking common reference points between the image and its stage position (marked as R1 and R2 in Fig. 2b). Microscope stage position was measured with an accuracy of ± 1 μm, but the accuracy of localization was limited by the resolution of the digital images, typically about ± 110 μm. To easily distinguish labeled structures, we marked the locations of labeled cells, fibers (anterograde, retrogradely labeled fibers are poorly labeled), and terminals with different symbols and colors. Figure 2b illustrates the same section as in 2a after microscopic plotting. Figure 2b shows more label detail than can be seen in the darkfield image shown in 2a. In this report, we plotted all of the results at high power using cross-polarized microscope illumination. Our plotting probably over-emphasizes regions of the light terminal label but locations of labeled structures are accurate.

Size bars on the figures were generated from the mounted sections after processing. The size bars do not account for the shrinkage of the tissue, which was often large and anisometric. Section levels were determined by matching relevant structures with stereotaxic atlas images (Berman 1968; Berman and Jones 1982). Due to variations in sectioning planes and wide atlas spacing, the levels should be considered approximate.

### Data availability

The corresponding author, Alan Gibson, has additional anatomical data that can be made available to researchers making specific requests.

## Results

### NRTP brainstem connections

**Fig. 3:**
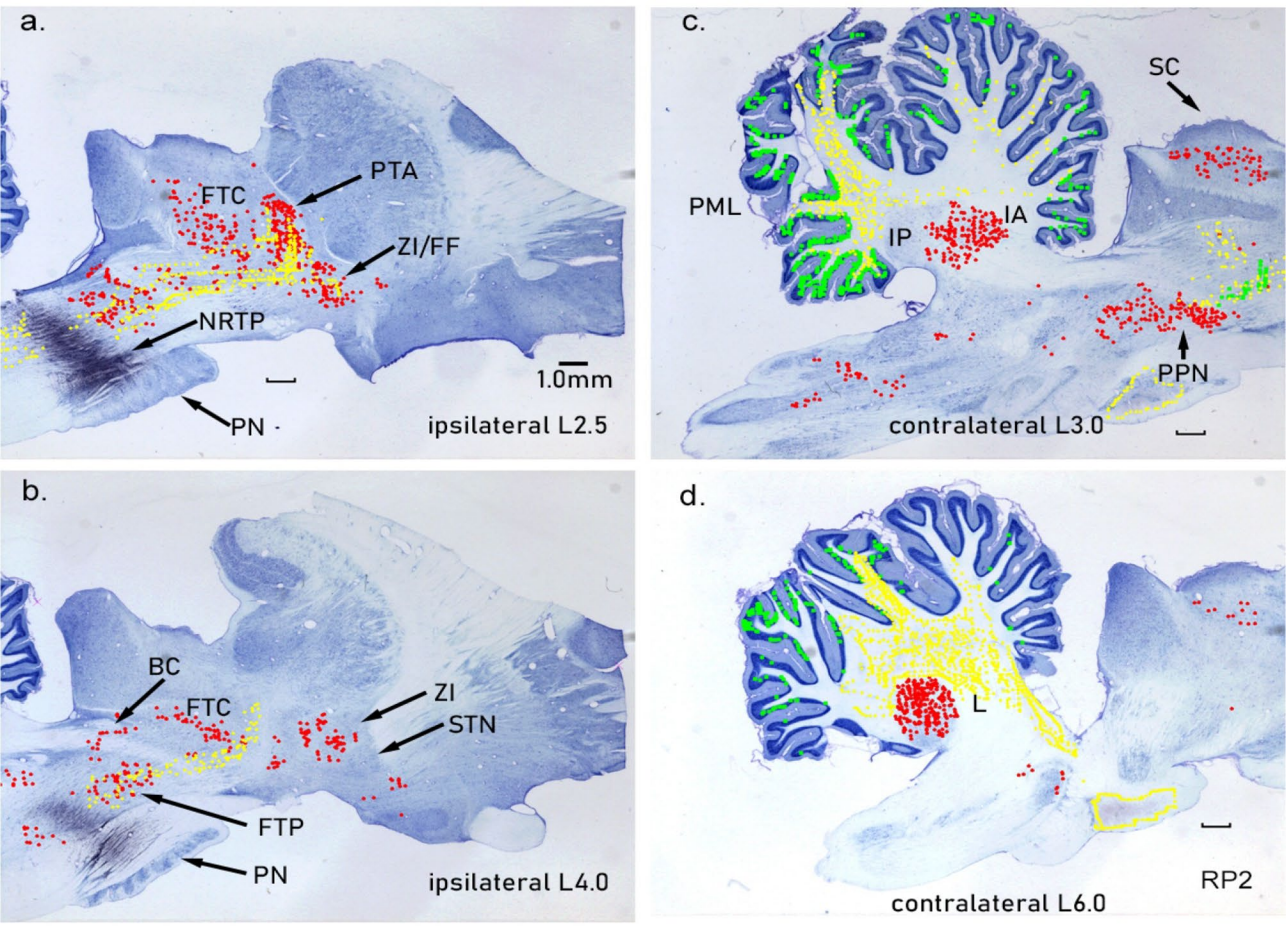
Connections of the cat nucleus reticularis tegmenti pontis (NRTP). Parasagittal brainstem sections, rostral to the right. Red demarcates labeled cells, green-labeled terminals and yellow anterogradely labeled fibers (the same labeling convention is used for all figures). Extensive input (red, labeled cells) to NRTP arises from various nuclei in the ipsilateral and contralateral brainstem as well as the cerebellar nuclei. Projections of NRTP extensively label mossy fiber glomeruli throughout the cerebellar cortex

We placed two WGA-HRP injections (6 nL, 1% WGA-HRP, each) into the cat NRTP (Fig. 3b). The injections were made at a stereotaxic laterality of 0.5 and 1.5 mm into cellular regions immediately dorsal to the pontine nuclei. Histology indicated that the label did not cross to the contralateral side of the brainstem. Pontine nuclei (PN) were not included in the injections, but the injection sites extended dorsally into the pontine tegmentum.

NRTP efferent projections were limited almost entirely to the cerebellum, although a light terminal label was present in the contralateral NRTP and in the pontine tegmentum contralateral to the injection. NRTP projects heavily to cerebellar cortex and labeled mossy fiber terminals were present in the cerebellar granular layers on both sides of the cerebellum. Terminal labeling was significantly greater in the cerebellar cortex contralateral to the NRTP injections (Fig. 3c, d). On the contralateral side, labeled mossy fiber terminals were present in the granular layer of the posterior lobe and were present in the folia of the vermis and hemisphere. Heavily labeled terminals were also present in the folia of the paramedian lobule and caudal vermis. In the contralateral hemisphere, the mossy fiber labeling was dense. The pattern of cerebellar cortical labeling is consistent with the findings of Gerrits and Voogd (1986), who used autoradiographic tracing to examine NRTP projections. Their study revealed NRTP mossy fiber projections to essentially the entire cerebellar cortex with the side contralateral to the injection being significantly stronger than the ipsilateral side—they reported no projections external to the cerebellum.

**Fig. 4:**
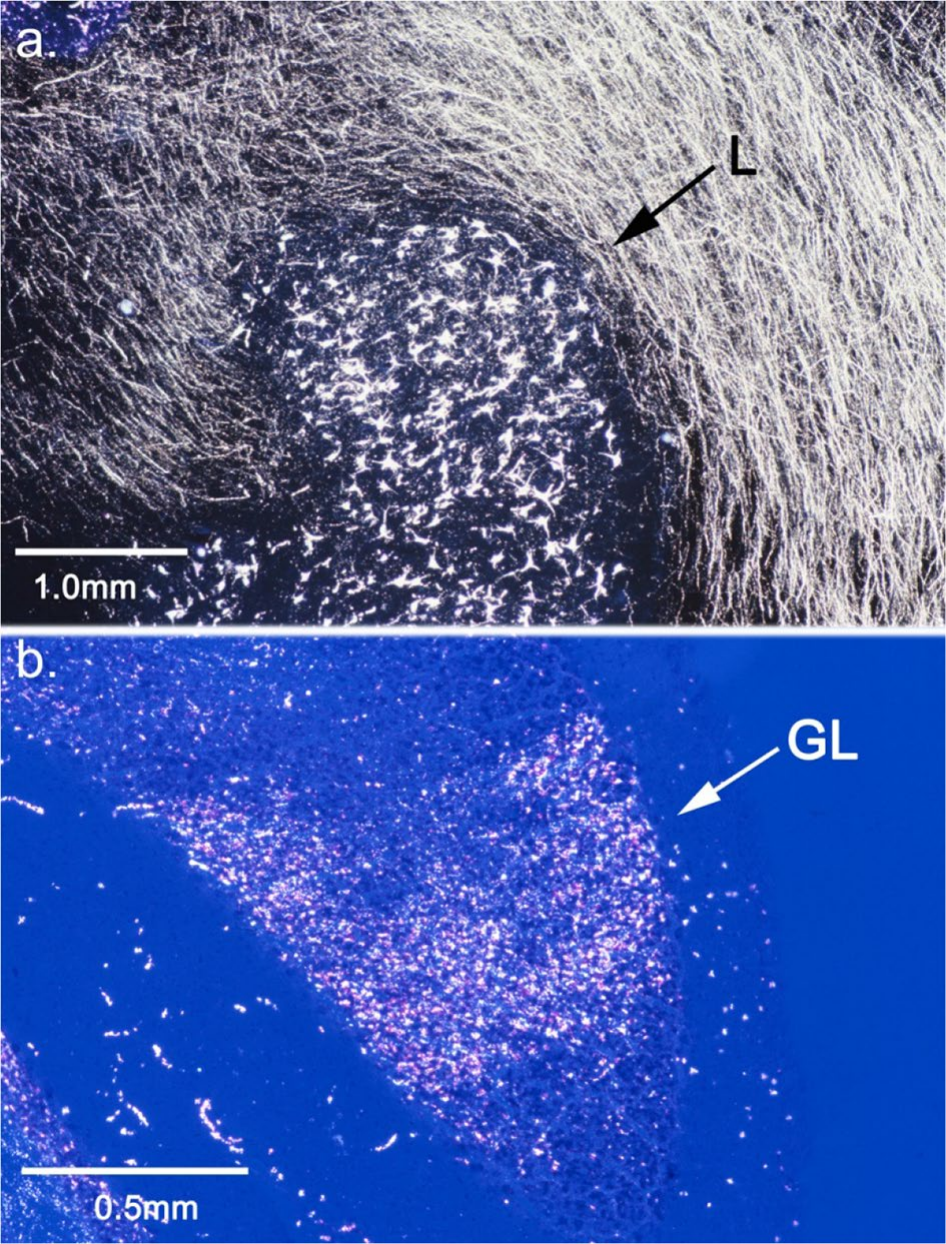
**a** Heavily labeled neurons in the lateral nucleus after NRTP injection (Fig. 3). **b** Labeled mossy fiber glomeruli in the cerebellar granular layer

Some regions of the cerebellar granular layer contained extremely dense labeled mossy fiber glomeruli, which can be seen in the higher-powered image shown in Fig. 4b. In the cerebellar cortex ipsilateral to the injection, terminals were labeled in the caudal vermis and many labeled fibers and some labeled terminals were present in the paramedian lobule and lateral regions of the hemisphere.

In contrast to the restricted efferent projections of NRTP, cellular labeling from the NRTP injections indicated that many regions project to NRTP. Cells in the cerebellar nuclei, especially the lateral nucleus (L, dentate), provide dense projections to NRTP. Contralateral to the injection, the lateral nucleus and anterior interposed nucleus (IA) contained many labeled cells (Fig. 3c, d). A higher-power photograph of labeled cells in the lateral nucleus is shown in Fig. 4a. Essentially every large cell is labeled, and a massive number of labeled mossy fibers can be seen arcing over the nucleus. The medial nucleus on both sides of the cerebellum contained labeled cells. Posterior interpositus was the only cerebellar nucleus that did not have a strong projection to NRTP (Fig. 3c). ZI and fields of Forel (referred to as ZI/FF since the border between regions is not clear) contained labeled cells on both sides with more on the side ipsilateral to the injection (Fig. 3a). STN was largely devoid of a label with only a few scattered labeled cells (Fig. 3b). We made additional WGA-HRP injections into brainstem areas containing retrogradely labeled cells to confirm projections to NRTP. The additional cases include the pedunculopontine nucleus (PPN), anterior pretectal nucleus (PTA), the midbrain tegmentum (FTC), superior colliculus (SC), and ZI/FF.

### Confirmation of projections to NRTP

#### PPN connections

**Fig. 5:**
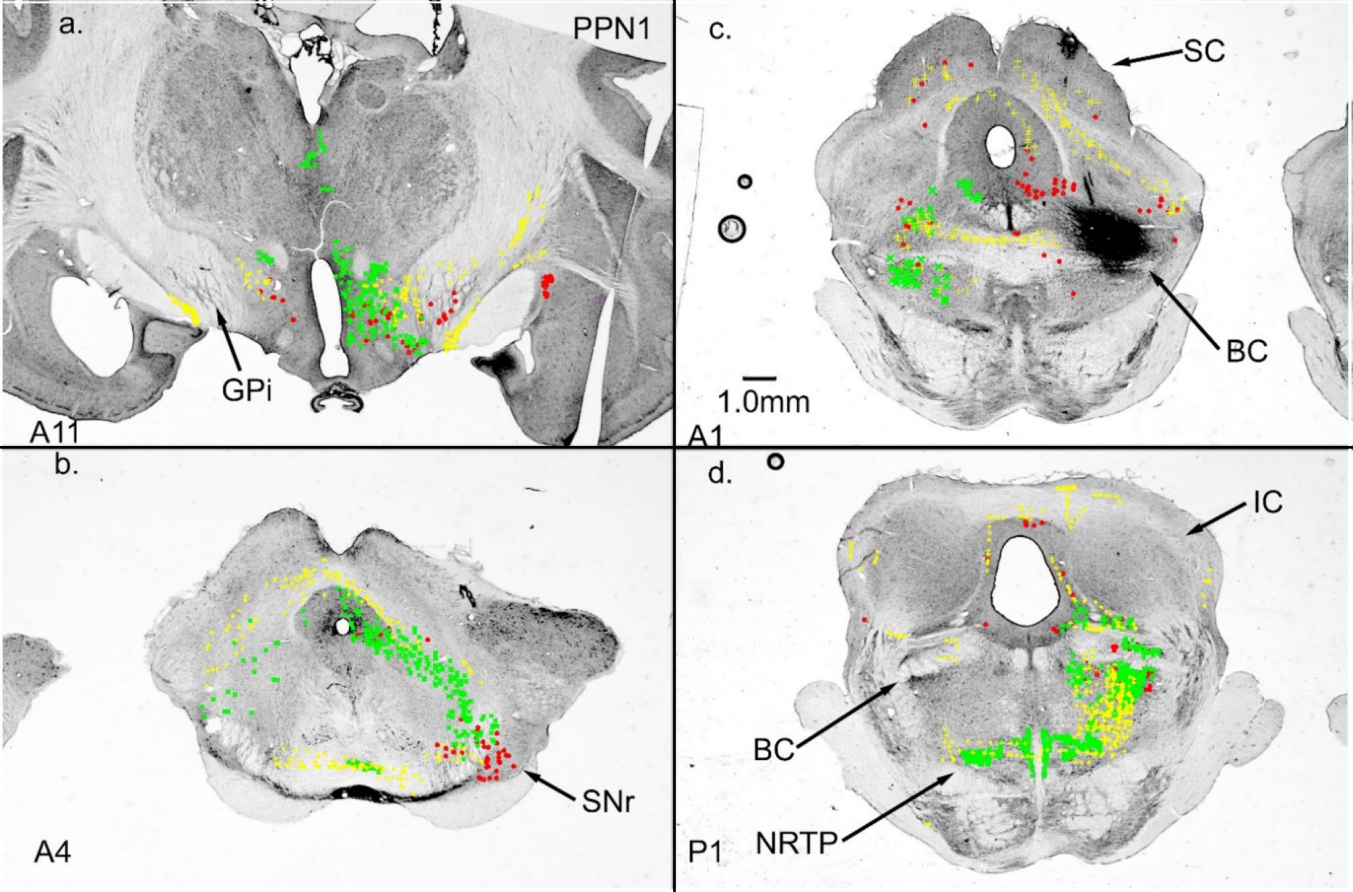
Connections of PPN dorsal to the brachium conjunctivum (BC). **a** Cells in GPi and hypothalamus projecting to PPN. **b** SNr cells projecting to PPN. **c** WGA-HRP injection site in PPN dorsal and into BC. **d** Bilateral terminal label in NRTP. The location of NRTP label suggests an overlap with labeling from case PPN2. Frontal Sects. (16nL of 1% WGA-HRP injected)

**Fig. 6:**
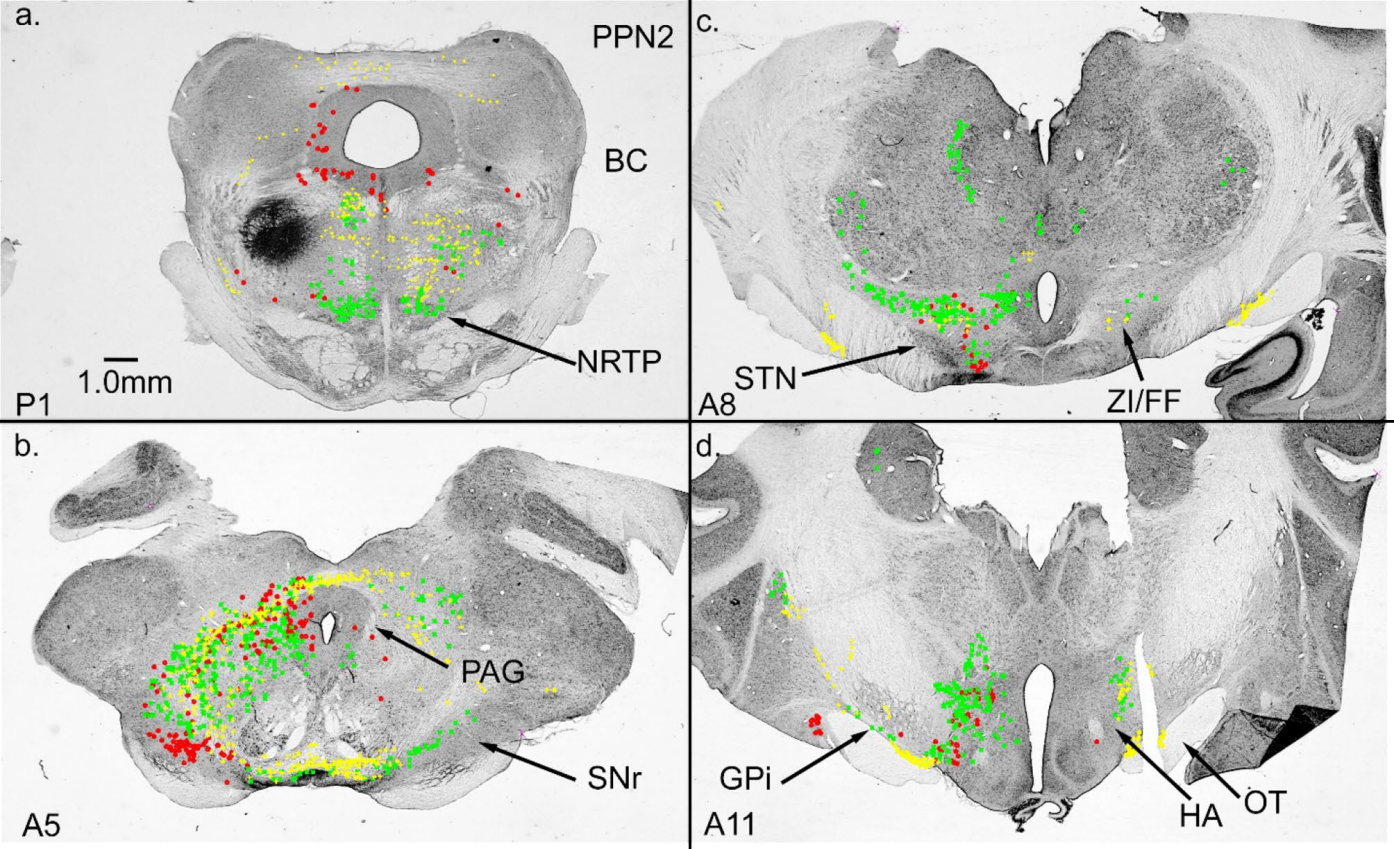
Frontal sections from case PPN2. **a** injection site (16nL, 1% WGA-HRP) in the BC and PPN ventral to BC. Bilateral terminal label in NRTP. **b** cells in SNr and in and around PAG provide input to this PPN region. **c** ZI/FF also provides input to the injected region of PPN, although STN has no connection. **d** few cells in GPi were labeled. As in PPN1, hypothalamic regions were well labeled

Our NRTP injection labeled cells surrounding the BC (Fig. 3b, c). We placed an injection at the border of the BC in two cases. One case (PPN1, Fig. 5c) was located at the dorsal border of BC at the level of BC decussation. The second case was located at the ventral border of BC at the level of the inferior colliculus (IC, Fig. 6a). Both injections produced bilateral terminal labeling over the anterior–posterior extent of NRTP, and labeled areas appeared to be similar for both cases (Figs. 5d, 6a). Both injections also produced many labeled cells in SNr (Figs. 5b, 6b), which receives input from GPi. However, labeling in other regions could be very different for the two injections.

Although not heavily labeled, case PPN1 resulted in retrogradely labeled cells in GPi throughout much of its AP extent (Fig. 5a). Case PPN2 produced very few labeled cells in GPi (Fig. 6d). Case PPN2 produced heavy terminal and cellular labeling in ZI/FF with no label in STN (Fig. 6c), whereas case PPN1 produced very little label in ZI/FF with a bilateral terminal label in STN. At the most anterior levels, both injections labeled many cells in the nucleus of the stria teminalis and nucleus of the anterior commissure.

Both PPN cases resulted in cellular labeling in the cerebellar nuclei, but the patterns of labeling were very different. PPN1 produced labeled cells in the caudal medial nucleus mainly on the side contralateral to the injection. Other cerebellar nuclei contained only a few scattered labeled cells. Case PPN2 produced many labeled cells in the nuclei with the lateral nucleus (L) and anterior interpositus (IA) being heavily labeled on the side ipsilateral to the injection. Posterior interpositus (IP) did not contain many labeled cells. The medial nucleus (M) was more heavily labeled on the side contralateral to the injection. Nuclear labeling might result from pickup by passing BC fibers, but different patterns of labeling from the PPN injections argue against this (1% WGA-HRP labels passing fibers poorly (Gibson et al. 1984), also, see fig. 10 in Pong et al. (2002). Clearly, the area surrounding the BC has regional specificity.

The PPN injections produced cellular and terminal labelling in the ponto-medullary tegmentum, with the side ipsilateral to the injection being heavier than the contralateral side. At cervical levels (lower levels were not processed), the ipsilateral ventral lateral funiculus contained scattered labeled fibers. The spinal gray contained a light terminal label largely confined to lamina VII. An occasional labeled cell was also present in the ipsilateral spinal gray. The current report focuses on potential basal ganglia input to the cerebellum. The cerebellum is involved in all movements and has a primary role in locomotion (see “Discussion”), which is clearly affected by basal ganglia disease. Certainly, the descending projections of PPN play some role in movement control and the contributions that different pathways make need to be further defined.

#### ZI/FF connections

**Fig. 7:**
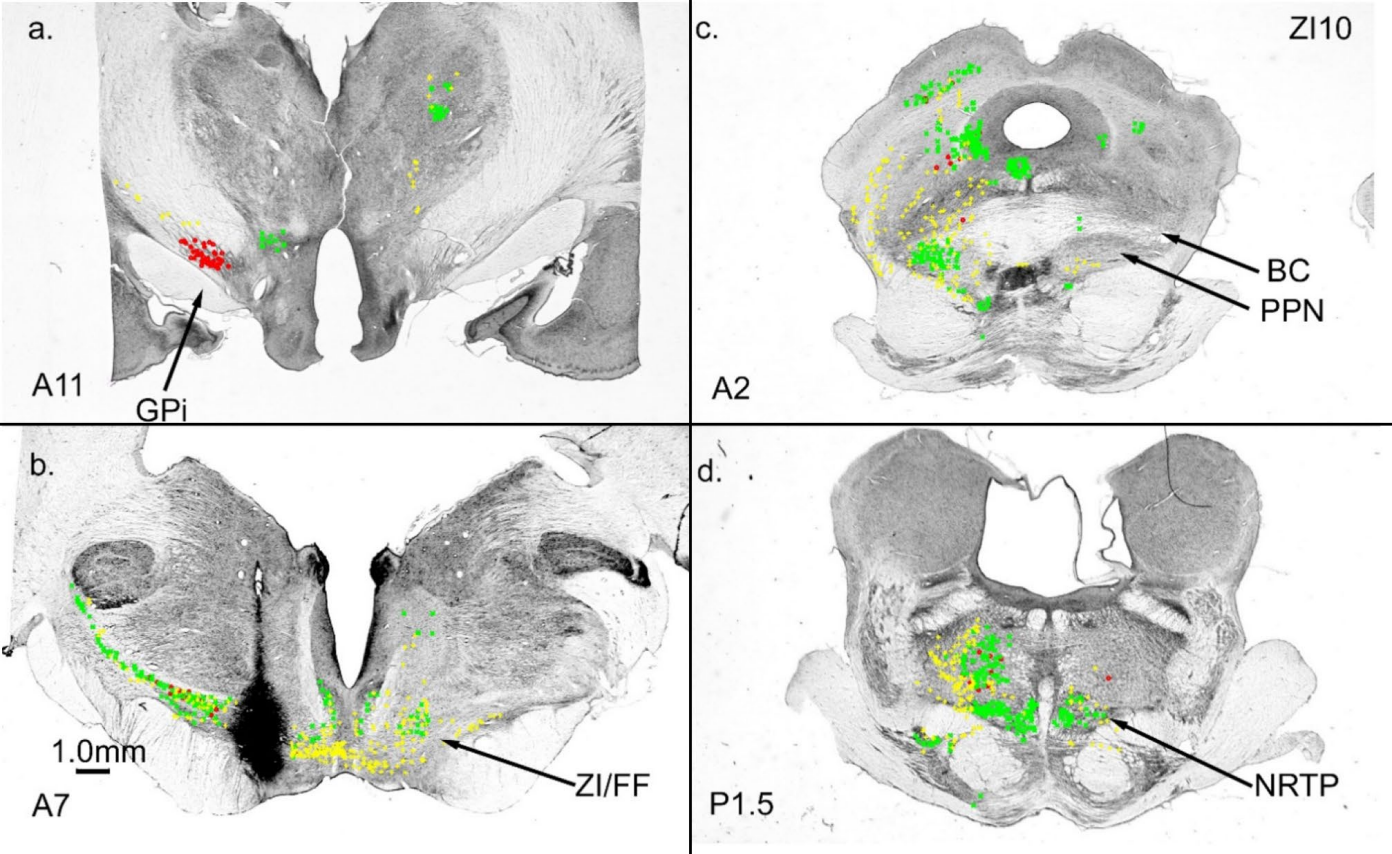
Connections of caudal ZI/FF. **a** labeled cells in GPi. **b** ZI/FF injection site just caudal to STN. **c** Terminal label in PPN. **d** Bilateral terminal label in NRTP and terminal and cellular label in the pontine tegmentum

**Fig. 8:**
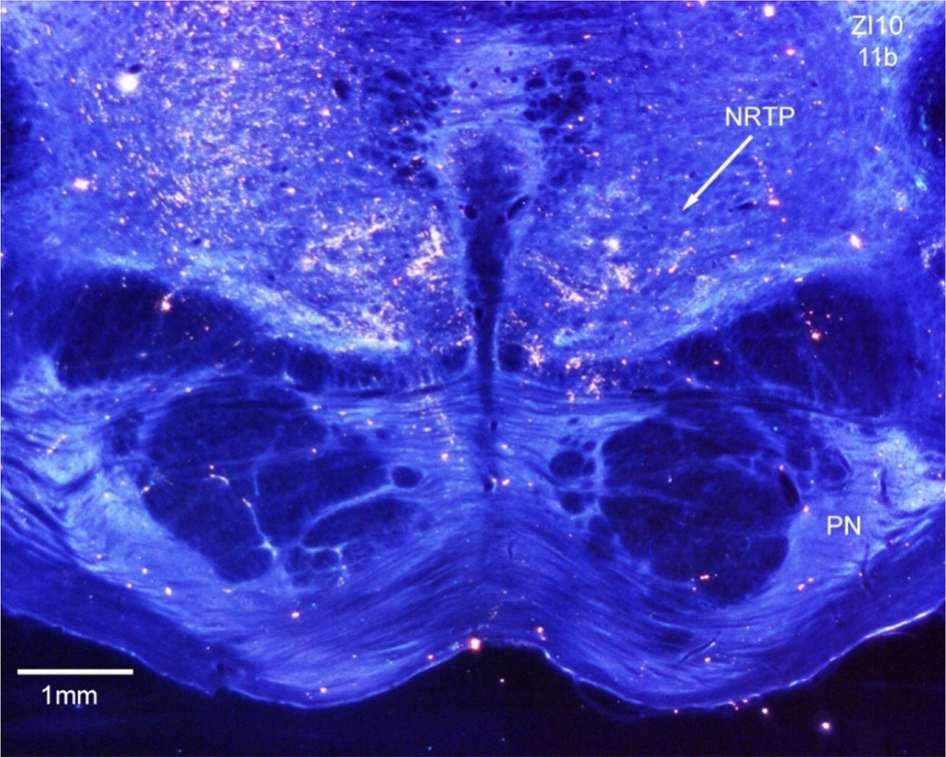
Terminal label in NRTP following injection into ZI/FF (Fig. 7b)

Our NRTP injection labeled a cluster of cells in ZI/FF dorsal and medial to the caudal pole of STN (Fig. 3a, b). An injection (ZI10, Fig. 7a, 14nL, 1%) into ZI/FF caudal to STN produced a bilateral terminal label in NRTP (Figs. 7d; 8). Terminal label was also present in the PPN (Fig. 7c). Labeled cells were present in GPi (Fig. 7a) through a large portion of its anterior–posterior extent. ZI/FF provides a relatively direct pathway from GPi to NRTP. Giolli et al. (2001) also reported a prominent projection from ZI to NRTP in the monkey.

#### FTC connections

**Fig. 9:**
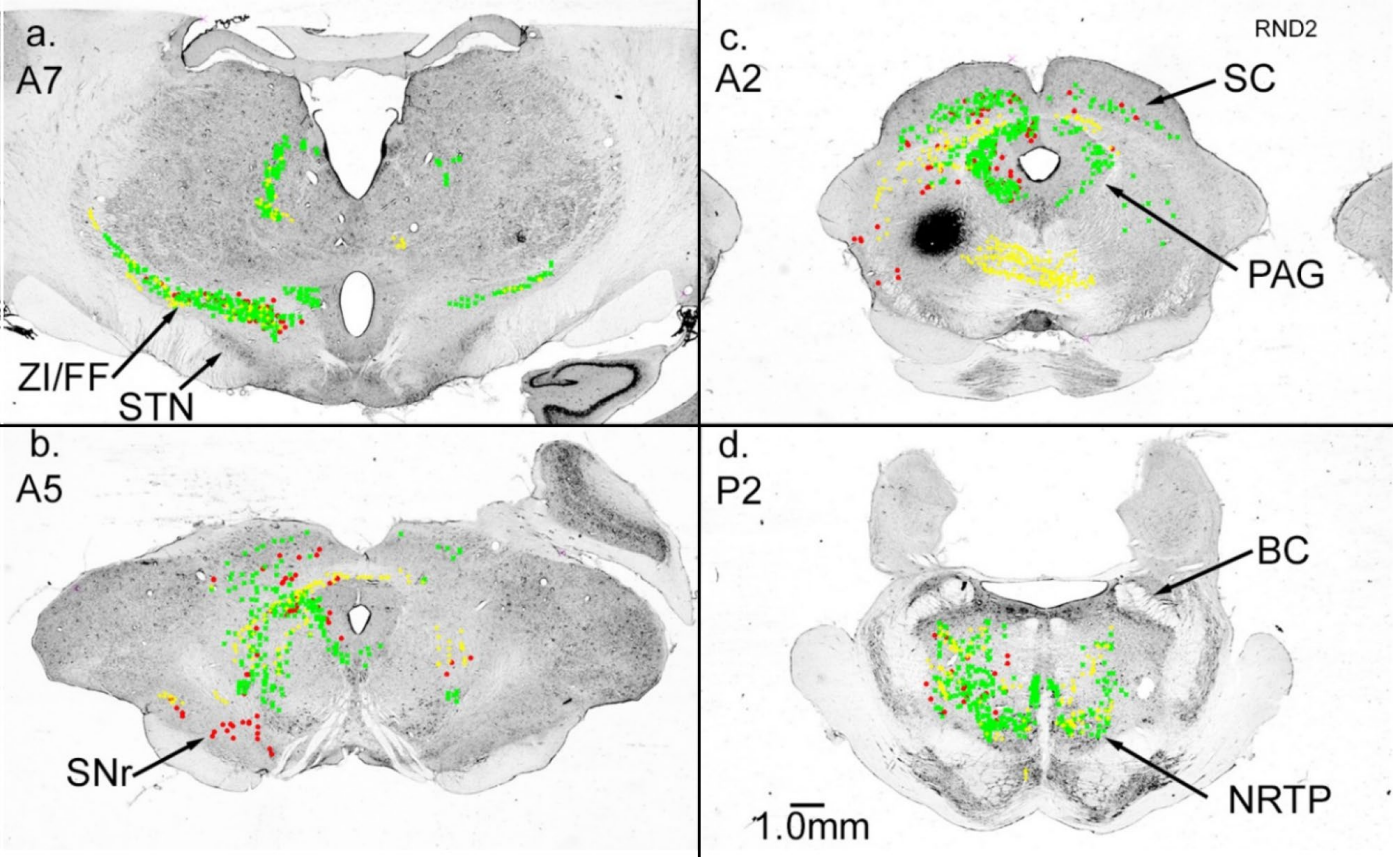
Labeling following a WGA-HRP injection (16nL, 1%) into caudal FTC (**c**). The injection labeled terminals and cells in ZI/FF, PAG, SC, and NRTP (**a**–**d**). SNr (**b**) contained many labeled cells but no labeled terminals. STN contained no label

Our NRTP injection labeled many cells in the midbrain central tegmental field (FTC) ipsilateral to the injection (Fig. 3a, b). Another cluster of tegmental cells projecting to NRTP was present dorsal and immediately lateral to the caudal pole of RNm. Placing a WGA-HRP injection (16 nl, 1%) in the corresponding region (Fig. 9c) confirmed a bilateral projection to NRTP (Fig. 9d). At the level of the injection site (9c) reciprocal projections were seen with the SC and lateral PAG. More rostrally, SNr contained a large number of retrogradely labeled cell (Fig. 9b). At the level of the STN, reciprocal connections were present in ZI/FF immediately dorsal as well as medial to STN (Fig. 9a).

#### PTA connections

**Fig. 10:**
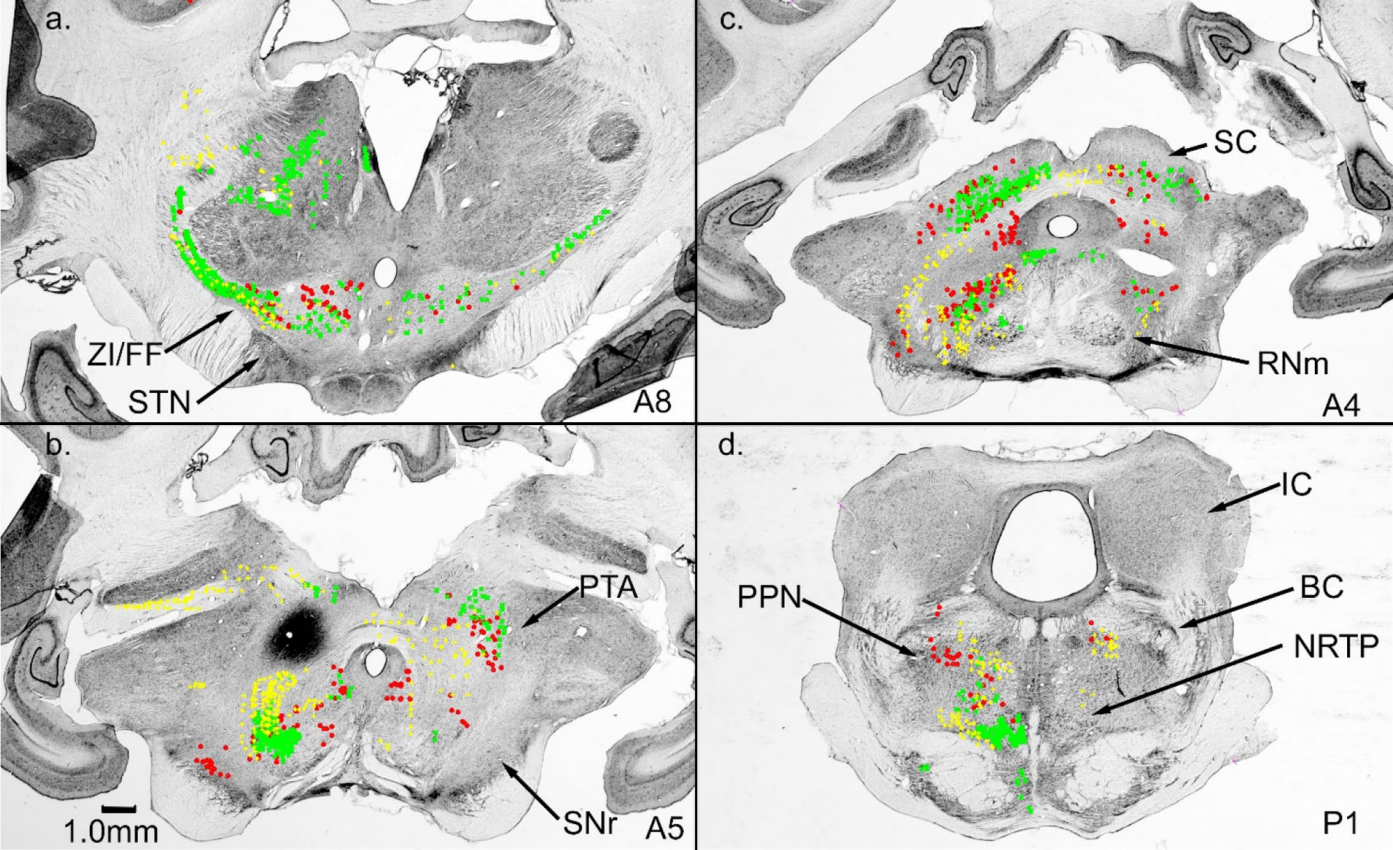
Connections of the anterior pretectal nucleus, PTA. **a** ZI/FF projections to and from PTA. **b** Injection site in PTA, labeled cells in SNr. **c** Heavy terminal and cellular labeling were present in SC and midbrain tegmentum dorsal to the red nucleus. **d** Terminal label in NRTP and labeled cells in PPN

Our NRTP injection resulted in well-labeled cells throughout the ipsilateral anterior pretectal nucleus (PTA, Fig. 3a). We placed a WGA-HRP injection (PTA 1; 12nL, 1%) into PTA (Fig. 10b). The injection produced terminal label in the ipsilateral NRTP (Fig. 10d), thereby confirming the projection from PTA to the NRTP. Labeled cells in the cerebellar nuclei exhibited a pattern that we did not observe from our other projections: The lateral nucleus (L) on both sides of the cerebellum contained many well-labeled cells with the side contralateral to the injection being more heavily labeled. Neither anterior nor posterior interpositus contained many labeled cells, but the caudal half of the medial nucleus on both sides of the cerebellum contained well-labeled cells.

SNr on the side of the PTA injection contained labeled cells (Fig. 10b). Other midbrain areas were heavily labeled with a terminal and cellular label. An area dorsal to RN contained many labeled cells as well as terminal labeling (Fig. 10c). The SC was well labeled with bilateral terminal and cellular labeling (Fig. 10c), with the side ipsilateral to the injection being more heavily labeled. The nucleus of the posterior commissure on the side contralateral to the injection contained labeled cells. ZI/FF was bilaterally labeled with a terminal and cellular label, with the side ipsilateral to the injection being heavier. At the level of the STN, ZI/FF contained labeled cells and terminals on both sides with the side ipsilateral to the injection being heavier. Regions dorsal to the STN were labeled (Fig. 10a), but the STN was not labeled.

#### SC connections

**Fig. 11:**
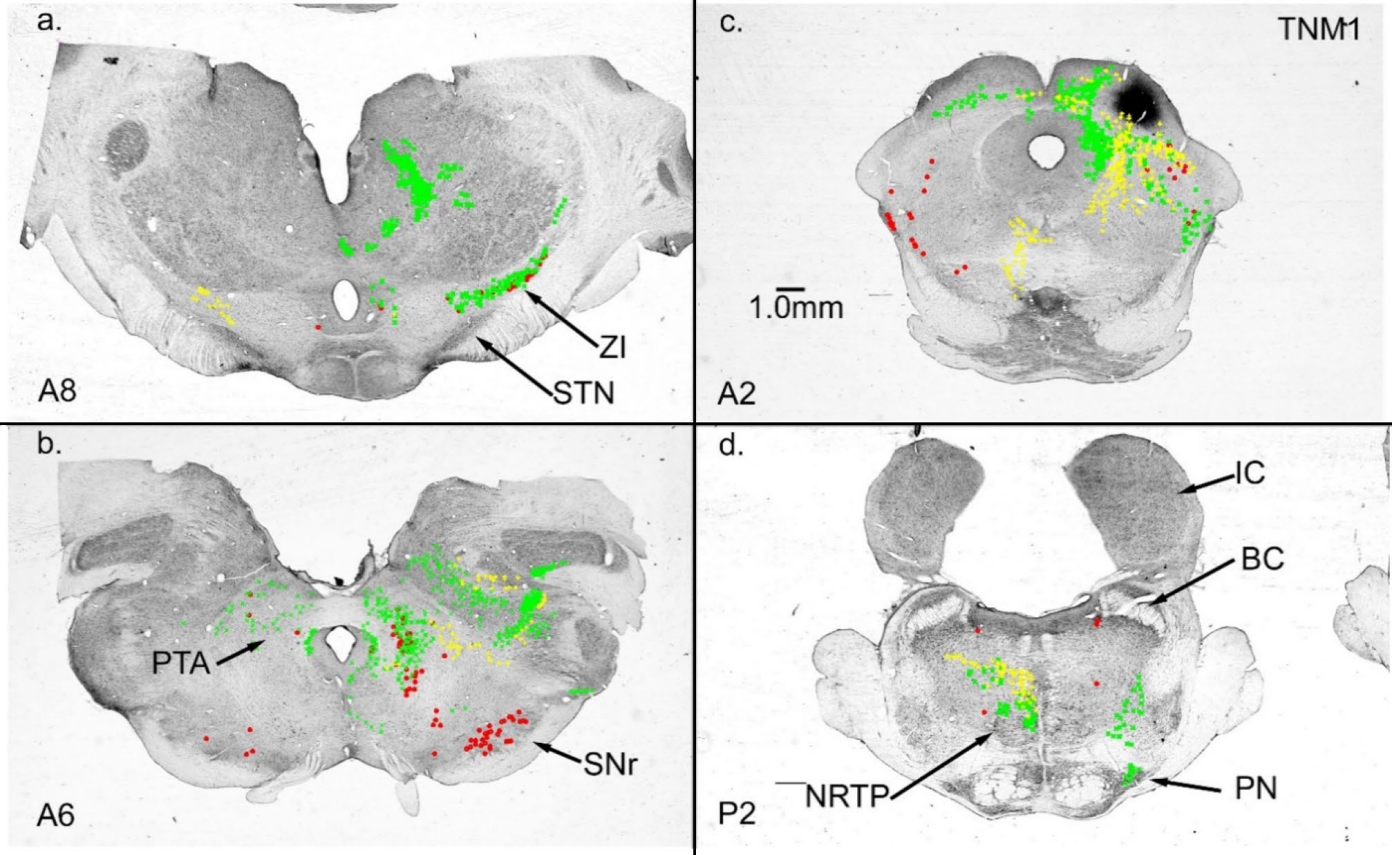
Connections of the superior colliculus, SC. **a** ZI projections to and from SC. **b** Cells in SNr projecting to SC. **c** WGA-HRP injection site in superior colliculus. **d** SC projection to NRTP and PN. Both ZI and SNr could relay basal ganglia output to NRTP via the SC. Frontal sections

The NRTP injection labeled neurons in the intermediate and deep layers of the contralateral SC. We placed a WGA-HRP injection (16nL, 1%) into SC (TNM1, Fig. 11c) to confirm the SC to NRTP pathway. The SC injection labeled axon terminals in NRTP contralateral to the injection (Fig. 11d. A small patch of terminal labeling was present also in the lateral pontine nuclei ipsilateral to the injection (PN, Fig. 11d). The SC injection labeled cells in the caudal half of the cerebellar medial nucleus on both sides, and cells in the lateral posterior interpositus and caudal lateral nucleus on the side ipsilateral to the injection. At the level of the exiting oculomotor nerve, the SC injection labeled many cells in ipsilateral SNr and both cells and terminals in the PTA and surrounding midbrain tegmentum (Fig. 11b). There were also some labeled cells in the contralateral SNr. More rostral at the level of the STN, there were labeled cells and terminals mainly in ZI ipsilateral to the injection (Fig. 11a). STN had no label.

**Fig. 12:**
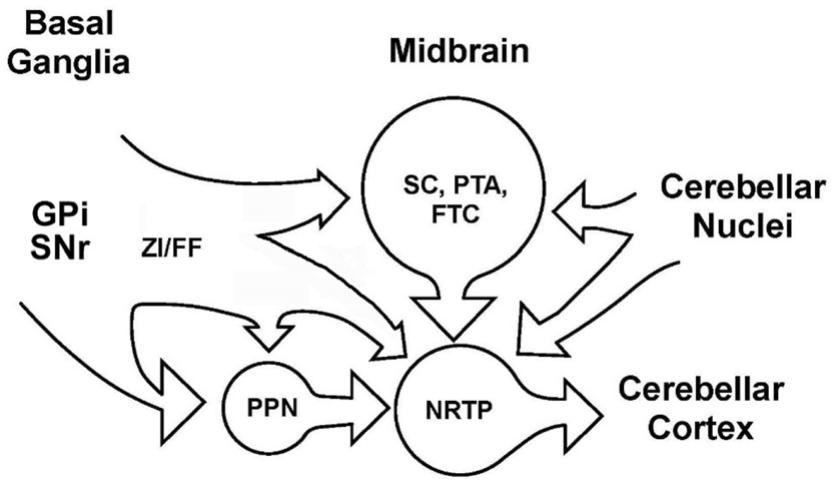
Simplified schematic of some of the pathways connecting basal ganglia output to the cerebellum via NRTP

In summary, many pathways involving regions of the sub thalamus, midbrain and pons are potential routes connecting basal ganglia output to the cerebellum via NRTP. We have highlighted some of the more direct pathways from GPi to NRTP, but midbrain anatomy is complex with extensive connections between regions. Figure 12 is a simplified schematic providing an overview illustrating how basal ganglia output could reach the cerebellum via the projections described in this report. The distribution of brainstem areas projecting to NRTP essentially mirrors the distribution of brainstem areas that receive input from the basal ganglia (Nauta 1979; McElvain et al. 2021).

Additionally, many other regions of the brain, such as the cerebral cortex, provide input to NRTP (Brodal 1980; Giolli et al. 2001).

## Discussion

### Interference with NRTP produces movement deficits

Our studies, as well as many others in the literature, indicate that several midbrain and pontine pathways carry basal ganglia output to NRTP. It is possible that these pathways mediate some of the motor symptoms associated with diseases of the basal ganglia.

Many studies using monkeys as subjects indicate that NRTP is critical to the control of eye movements. In humans, diseases of the basal ganglia produce a broad range of eye movement deficits (Pretegiani and Optican 2017). Even relatively weak stimulation of NRTP can disrupt the smooth pursuit eye movements (Yamada et al. 1996), which can be abnormal in patients with Parkinson’s disease (Frei 2021). Additionally, the inactivation of NRTP slows and reduces amplitudes of saccadic eye movements (Kaneko and Fuchs 2006). Wurtz and Hikosaka (1986) outlined a basal ganglia pathway for eye movement control that involved SNr projections to the SC and hypothesized that similar circuits may be involved in other movements, which would include SNr pathways to NRTP. Our injection in NRTP labeled mossy fiber terminals over the entire extent of the paramedian lobule (PML, Fig. 3), which contains a somatotopy of the entire body (Snider and Eldred 1952). The extensive mossy fiber labeling in the cerebellar cortex after NRTP injection indicates that NRTP is involved in the control of the musculature of the entire body, which is supported by the fact that NRTP receives input from all regions of the motor cortex (Brodal and Brodal 1971).

In contrast to eye movement studies, there have been only a few studies investigating NRTP participation in limb movements. Some cells in NRTP discharge during limb movements (Matsunami 1987), and in the decorticate cat many NRTP cells fire rhythmically during spontaneous locomotion (Zangger and Schultz 1978).

Additional evidence that NRTP may mediate basal ganglia output comes from a series of studies of the rat NRTP. Lesions of the NRTP in the rat induce rapid forward locomotion (festination) as well as balance deficits (Cheng et al. 1981; Brudzynski and Mogenson 1984), which are frequent symptoms of Parkinson’s disease. Haloperidol, a dopamine receptor blocker (D2 receptors), applied systemically in normal rats produces akinesia, but does not block festination induced by NRTP lesions (Chesire et al. 1983). Additionally, damage to the rat NRTP or inactivation with drugs can relieve akinesia induced by systemically administered haloperidol and induce festination (Chesire et al. 1984). Festination and gait abnormalities in monkeys have also been reported following bilateral lesions involving the cerebellar dentate nucleus (Botterell 1938). Together, the findings add support to the hypothesis that movement disorders associated with diseases of the basal ganglia could be mediated via NRTP projections to the cerebellum.

### Descending cerebellar projections

A pathway from the basal ganglia to the cerebellum via NRTP could explain some puzzling observations of Parkinson’s disease. For example, lesions of the thalamus can be effective for relieving tremor but are not effective for relieving other symptoms such as bradykinesia and akinesia. In fact, lesions, even very large ones, of cerebellar-receiving regions of the thalamus have little effect on limb movements in monkeys, cats, and humans (Canavan et al. 1989; Fabre-Thorpe and Levesque 1991; Bastian and Thach 1995; Duval et al. 2006). Still, lesions of the lateral cerebellum including the lateral nucleus produce severe movement deficits (Botterell 1938; Carpenter and Stevens 1957). The lateral nucleus projects to the motor cortex via brachial fibers projecting to the thalamus. Interrupting brachial fibers anterior to or in the red nucleus of the monkey produces tremor but no limb ataxia. In contrast, interrupting brachial fibers caudal to the red nucleus produces severe limb ataxia (Carrea and Mettler 1955; Carpenter 1956). Descending fibers of the brachium separate from the ascending fibers caudal to the red nucleus, and limb ataxia is probably due to disruption of output to NRTP as well as other brainstem areas.

Although the destruction of cerebellothalamic projections to the cortex minimally disrupts limb movements, output from the cortex is important for limb control, especially output reaching the midbrain and pons. It is generally believed that cortical control of movement is achieved by projections of motor cortical areas to the spinal cord via the pyramidal tract, however, section of the pyramidal tract of the cat does not disrupt general movements or movements produced by cortical stimulation (Nieoullon and Gahery 1978). Lawrence and Kuypers (Lawrence and Kuypers 1968) interrupted cortical output to the lower brainstem and spinal cord of monkeys by sectioning the pyramids caudal to the pons. The monkeys were able to reach for food within 24 to 48 h after recovery from anesthesia, and, within a few days, they could stand, walk, and climb. The monkeys could reach and grasp food with the whole hand, but they had a long-lasting deficit in controlling fine finger movements. In contrast, a much stronger deficit in limb movements results following a section of the cerebral peduncle (Bucy et al. 1966). The monkeys immediately had paralysis of limb movements, and several months of recovery were needed before they could walk and climb. Forelimbs were more affected than hindlimbs, and some of the monkeys had ataxic limb movements and never recovered the ability to pick up food. It is likely that the greater deficit produced by section of the cerebral peduncle is due to the interruption of cortical input to the midbrain and pons. The cerebellum, via pontine nuclei and NRTP, receives a massive input from the motor cortex as well as additional cortical areas (Brodal and Brodal 1971; Glickstein et al. 1985; Giolli et al. 2001), and input to NRTP emphasizes forelimb areas of the motor cortex (Matsuyama and Drew 1997). Therefore, the much greater movement deficits seen with lesions of the cerebral peduncle versus the medullary pyramidal tract may be due to interruption of input to the cerebellum. Finger use deficits after the pyramidal tract section may reflect cortical importance for movements requiring immediate sensory feedback (Wall 1970; Wall and Noordenbos 1977; Favorov et al. 1988).

Projections from the lateral cerebellar nucleus and interpositus to contralateral NRTP constitute a major component of descending brachial fibers (Miller and Strominger 1977; Asanuma et al. 1983). Our injection into NRTP demonstrated an extremely heavy input from cerebellar nuclei, especially the contralateral lateral nucleus. Additionally, many of the areas receiving basal ganglia output that project to NRTP also receive input from cerebellar nuclei with heavy input from the lateral nucleus. Our injection into the ventral PPN of the cat produced many labeled cells in the lateral nucleus and anterior interpositus. Lateral nucleus (dentate) terminations in the PPN of the squirrel monkey have been demonstrated by Hazrati and Parent (Hazrati and Parent 1992). Our ZI/FF and PTA injections heavily labeled cells within the lateral nucleus. Any or all these pathways could allow cerebellar output to affect movements via input to NRTP.

### Behavioral relations of brainstem regions projecting to NRTP

Several midbrain regions that receive BG input and project to NRTP produce a variety of behaviors when stimulated electrically or chemically. Stimulation of the PPN and regions dorsal to PPN produces locomotion in the decerebrate cat, and stimulation immediately ventral to PPN produces atonia (Takakusaki et al. 2016). In the intact cat, electrical stimulation of the midbrain tegmentum (FTC) can elicit basic behaviors necessary for survival such as attack or flight (Sheard and Flynn 1967). Such behaviors require complex sensory guidance and rapid limb control. Neither the PAG nor FTC have spinal projections to support rapid movements, although it is thought that midbrain projections to large cells in the ponto-medullary reticular formation could mediate rapid movements. However, the destruction of these cells in the cat brainstem with kainic acid has been reported to have little effect on limb movements (Sastre et al. 1981). Not only is the cerebellum well suited to integrate sensory information with motor control, but its output pathways to spinal levels are some of the fastest in the nervous system. In the cat, stimulation of LVN produces EPSPs in cervical motoneurons in ~ 1 ms (Udo et al. 1979) and lumbar motoneurons in ~ 2 ms (Grillner and Hongo 1972; Matsuyama and Jankowska 2004).

Only a few cells in the cat midbrain periaqueductal gray (PAG) project directly to NRTP, but PAG has extensive connections with FTC and PPN (Shaikh et al. 1987) which project to NRTP (Figs. 6b, 9c). Electrical or chemical activation of the cat PAG elicits defensive rage and predatory attack (Gregg and Siegel 2003; Bhatt et al. 2008). Again, these are behaviors that require rapid guided limb movements. Chemical or optogenetic excitation of the cat PAG can also produce flight or immobility (Zhang et al. 1990; Tovote et al. 2016). Akinesia and freezing are cardinal motor symptoms of PD (Rahimpour et al. 2021). In the mouse, optogenetic activation and inactivation of RtTg (NRTP) modify the startle response (Guo et al. 2021), which is another defensive reaction. Optogenetic modulation of vermal Purkinje cells also modifies aggressive behavior (Jackman et al. 2020). Therefore, the cerebellum is at least involved in behavioral responses associated with the PAG and may mediate them. Behaviors elicited by PAG stimulation are complex and require rapid access to motor areas as well as coordination with autonomic actions, which may be achieved via cerebellar processing (Bandler and Shipley 1994; Fujita et al. 2020; Romano et al. 2020).

### Deep brain stimulation could influence cerebellar pathways

Electrical stimulation of the subthalamic nucleus (STN) is an established clinical therapy for reducing motor deficits associated with PD (Benabid et al. 2009; Hariz and Blomstedt 2022), and the STN is considered a potential element in BG connections to the cerebellum. None of our cases labeled more than a few cells in STN, and it is likely that the effects of STN stimulation are mediated by its projections to GPi and SNr. It is possible that STN stimulation activates neurons in the surrounding ZI/FF, which project to NRTP. Stimulation of ZI/FF improves PD motor symptoms, perhaps more effectively than STN stimulation (Plaha et al. 2006; Ossowska 2020; Stenmark Persson et al. 2022). Additionally, STN receives a projection from PPN, and STN stimulation may antidromically activate PPN neurons that also project to NRTP. In any case, multiple pathways exist that could allow STN stimulation to modify activity in NRTP and influence cerebellar action.

Using retrograde viral tracing methods in the cebus monkey, Bostan et al. (2010) reported a disynaptic projection from the STN to the cerebellar cortex via a projection to pontine nuclei (PN). Although we agree that the cerebellum receives input from the basal ganglia, at least in other species, a major projection from STN to pontine nuclei is unlikely. Efferent projections of the STN have been extensively studied in monkeys, cats, and rats (Nauta and Cole 1978; McBride and Larsen 1980; Carpenter et al. 1981; Edley and Graybiel 1983; Aas 1989; Mihailoff et al. 1989; Smith et al. 1990; Giolli et al. 2001), and none of the studies reported an STN projection to pontine nuclei. The most direct pathways from basal ganglia to the cerebellum are via GPi and SNr to PPN and ZI/FF, which provide input to NRTP. BG outputs to other brainstem regions add many additional, but less direct, pathways for basal ganglia output to reach the cerebellum via NRTP.

### Descending cerebellar pathways for rapid motor control

Three relatively direct cerebellar pathways to motor neurons in the spinal cord are interpositus projections to the magnocellular red nucleus (RNm), spinal projections of the superior colliculus (SC), and Purkinje cell and medial nucleus projections to the lateral vestibular nucleus (LVN). We believe that the LVN pathway most likely mediates limb movements. The SC projects to motoneuron pools in the upper cervical cord that innervate neck musculature (Shinoda et al. 1996). The RNm projects selectively to motor neurons innervating digit muscles (McCurdy et al. 1987; Robinson et al. 1987; Holstege et al. 1988), and behavioral studies indicate that RNm is important for finger movements related to grasping but not for the primary control of movements involving proximal limb muscles (Gibson et al. 1985, 1996; van Kan et al. 1994). The LVN projects to spinal levels via the lateral vestibulospinal tract (LVST). The LVST is an ipsilateral spinal pathway with a strong influence on contralateral as well as ipsilateral proximal limb musculature (Grillner and Hongo 1972; Hongo et al. 1975; Maeda et al. 1975; Shinoda et al. 1986; Matsuyama and Jankowska 2004). LVST is important for the control of locomotion and balance (McCall et al. 2017; Murray 2018).

Our NRTP injection labeled many cells in the cerebellar nuclei including the medial nuclei. The medial nuclei have bilateral projections to LVN, which may help coordinate across the body. The crossed projection terminates in the LVN (and NRTP) via fibers (uncinate fasciculus) in the cerebellar white matter (Ruggiero et al. 1977; Asanuma et al. 1983). Stimulation of the crossing fibers in the decerebrate cat induces locomotion (Mori et al. 1999, 2000). Fictive locomotion in the decerebrate cat depends upon an intact cerebellum, and rhythmic discharge of brainstem reticular cells can only be partially restored in the decerbellate preparation by mechanical movement of the hindlimbs (Orlovsky 1972), which would excite afferent input to the cells. Cooling of a relatively small area of the paravermal cortex in the decerebrate cat greatly distorts the pattern of fictive locomotion (Udo et al. 1979). PD patients have problems with limb movements including locomotion and balance; it is very likely that these deficits could be produced by disturbed cerebellar input to LVN.

A major problem with the hypothesis that LVN mediates limb movements is that it does not explain how the cerebellar lateral nucleus affects locomotion and other limb movements, since the lateral nucleus does not project to LVN (Carleton and Carpenter 1983; Pogossian and Fanardjian 1992; Voogd 2016). However, the lateral nucleus provides a large input to NRTP as well as to midbrain nuclei that project to NRTP. NRTP occupies a relatively small area and receives afferents from an incredibly large number of brain regions, and at least some afferent projections overlap within NRTP (Brodal and Brodal 1971). In the rat, projections from the lateral nucleus include most of NRTP (Angaut et al. 1985). A very small tracer injection into the rat NRTP produces bands of labeled mossy fibers in the cerebellar cortex, which include the vermal as well as the hemispheric cortex (Serapide et al. 2002). In the monkey, lateral nucleus projections to NRTP appear to be restricted to the central third of NRTP (Asanuma et al. 1983). Injections that include the central third of cat NRTP label cerebellar cortical areas that project to LVN (Gerrits and Voogd 1986). It is possible that NRTP projections to paravermal cortex allow lateral nucleus output to influence LVN activity, but this needs to be examined experimentally.

## Concluding remarks

The current report shows that NRTP provides a bridge between the basal ganglia and cerebellum. Brain regions in addition to those that we demonstrate may also use NRTP to modify cerebellar action (Glickstein et al. 1985; Giolli et al. 2001). Much of movement control by the cerebellum is expressed via vestibular pathways (McCall et al. 2017). This may be because early in vertebrate evolution, as sea animals adapted to land, the vestibular system was primarily important for maintaining visual and postural stability during movement. To be effective, the vestibular system required pathways that allowed rapid control of body musculature including limbs. As evolution progressed, additional functions such as targeting and grasping objects or making complex swimming maneuvers required rapid control of limb movements, especially the forelimb. To guide these movements, additional regions of the brain, such as the cerebral cortex and basal ganglia, needed access to existing vestibular pathways. NRTP, as well as the pontine nuclei, provide a cerebellar interface that allows other brain regions to control and modify movements produced via vestibular pathways.

## References

[CR1] Aas JE (1989). Subcortical projections to the pontine nuclei in the cat. J Comp Neurol.

[CR2] Angaut P, Cicirata F, Panto MR (1985). An autoradiographic study of the cerebellopontine projections from the interposed and lateral cerebellar nuclei in the rat. J Hirnforsch.

[CR3] Asanuma C, Thach WT, Jones EG (1983). Brainstem and spinal projections of the deep cerebellar nuclei in the monkey, with observations on the brainstem projections of the dorsal column nuclei. Brain Res.

[CR4] Aziz TZ, Davies L, Stein J, France S (1998). The role of descending basal ganglia connections to the brain stem in parkinsonian akinesia. Br J Neurosurg.

[CR5] Bandler R, Shipley MT (1994). Columnar organization in the midbrain periaqueductal gray: modules for emotional expression?. Trends Neurosci.

[CR6] Bastian AJ, Thach WT (1995). Cerebellar outflow lesions: a comparison of movement deficits resulting from lesions at the levels of the cerebellum and thalamus. Ann Neurol.

[CR7] Benabid AL, Chabardes S, Mitrofanis J, Pollak P (2009). Deep brain stimulation of the subthalamic nucleus for the treatment of Parkinson's disease. Lancet Neurol.

[CR8] Berman AL (1968). The brain stem of the cat; a cytoarchitectonic atlas with stereotaxic coordinates.

[CR9] Berman AL, Jones EG (1982). The thalamus and basal telencephalon of the cat: a cytoarchitectonic atlas with stereotaxic coordinates.

[CR10] Bhatt S, Bhatt R, Zalcman SS, Siegel A (2008). Role of IL-1 beta and 5-HT2 receptors in midbrain periaqueductal gray (PAG) in potentiating defensive rage behavior in cat. Brain Behav Immun.

[CR11] Bostan AC, Strick PL (2010). The cerebellum and basal ganglia are interconnected. Neuropsychol Rev.

[CR12] Bostan AC, Strick PL (2018). The basal ganglia and the cerebellum: nodes in an integrated network. Nat Rev Neurosci.

[CR13] Botterell EH, Fulton JF (1938). Functional localization in the cerebellum of primates. III. Lesions of the hemispheres (neocerebellum). J Comp Neurol.

[CR14] Brodal P (1980). The projection from the nucleus reticularis tegmenti pontis to the cerebellum in the rhesus monkey. Exp Brain Res.

[CR15] Brodal A, Brodal P (1971). The organization of the nucleus reticularis tegmenti pontis in the cat in the light of experimental anatomical studies of its cerebral cortical afferents. Exp Brain Res.

[CR16] Brudzynski SM, Mogenson GJ (1984). The role of the nucleus reticularis tegmenti pontis in locomotion: a lesion study in the rat. Brain Res Bull.

[CR17] Bucy PC, Ladpli R, Ehrlich A (1966). Destruction of the pyramidal tract in the monkey. The effects of bilateral section of the cerebral peduncles. J Neurosurg.

[CR18] Caligiore D, Pezzulo G, Baldassarre G (2017). Consensus paper: towards a systems-level view of cerebellar function: the interplay between cerebellum, basal ganglia, and cortex. Cerebellum.

[CR19] Canavan AG, Nixon PD, Passingham RE (1989). Motor learning in monkeys (Macaca fascicularis) with lesions in motor thalamus. Exp Brain Res.

[CR20] Carleton SC, Carpenter MB (1983). Afferent and efferent connections of the medial, inferior and lateral vestibular nuclei in the cat and monkey. Brain Res.

[CR21] Carpenter MB (1956). A study of the red nucleus in the rhesus monkey; anatomic degenerations and physiologic effects resulting from localized lesions of the red nucleus. J Comp Neurol.

[CR22] Carpenter MB, Stevens GH (1957). Structural and functional relationships between the deep cerebellar nuclei and the brachium conjunctivum in the rhesus monkey. J Comp Neurol.

[CR23] Carpenter MB, Carleton SC, Keller JT, Conte P (1981). Connections of the subthalamic nucleus in the monkey. Brain Res.

[CR24] Carrea RM, Mettler FA (1955). Function of the primate brachium conjunctivum and related structures. J Comp Neurol.

[CR25] Cheng JT, Schallert T, De Ryck M, Teitelbaum P (1981). Galloping induced by pontine tegmentum damage in rats: a form of "Parkinsonian festination" not blocked by haloperidol. Proc Natl Acad Sci U S A.

[CR26] Chesire RM, Cheng JT, Teitelbaum P (1983). The inhibition of movement by morphine or haloperidol depends on an intact nucleus reticularis tegmenti pontis. Physiol Behav.

[CR27] Chesire RM, Cheng JT, Teitelbaum P (1984). Reversal of akinesia and release of festination by morphine or GABA applied focally to the nucleus reticularis tegmenti pontis. Behav Neurosci.

[CR28] Cragg B (1980). Preservation of extracellular space during fixation of the brain for electron microscopy. Tissue Cell.

[CR29] Duval C, Panisset M, Strafella AP, Sadikot AF (2006). The impact of ventrolateral thalamotomy on tremor and voluntary motor behavior in patients with Parkinson's disease. Exp Brain Res.

[CR30] Edley SM, Graybiel AM (1983). The afferent and efferent connections of the feline nucleus tegmenti pedunculopontinus, pars compacta. J Comp Neurol.

[CR31] Fabre-Thorpe M, Levesque F (1991). Visuomotor relearning after brain damage crucially depends on the integrity of the ventrolateral thalamic nucleus. Behav Neurosci.

[CR32] Favorov O, Sakamoto T, Asanuma H (1988). Functional role of corticoperipheral loop circuits during voluntary movements in the monkey: a preferential bias theory. J Neurosci.

[CR33] Frei K (2021). Abnormalities of smooth pursuit in Parkinson's disease: a systematic review. Clin Park Relat Disord.

[CR34] French IT, Muthusamy KA (2018). A review of the pedunculopontine nucleus in Parkinson's disease. Front Aging Neurosci.

[CR35] Fujita H, Kodama T, du Lac S (2020). Modular output circuits of the fastigial nucleus for diverse motor and nonmotor functions of the cerebellar vermis. Elife.

[CR36] Gerrits NM, Voogd J (1986). The nucleus reticularis tegmenti pontis and the adjacent rostral paramedian reticular formation: differential projections to the cerebellum and the caudal brain stem. Exp Brain Res.

[CR37] Gerrits NM, Voogd J (1987). The projection of the nucleus reticularis tegmenti pontis and adjacent regions of the pontine nuclei to the central cerebellar nuclei in the cat. J Comp Neurol.

[CR38] Gibson AR, Hansma DI, Houk JC, Robinson FR (1984). A sensitive low artifact TMB procedure for the demonstration of WGA-HRP in the CNS. Brain Res.

[CR39] Gibson AR, Houk JC, Kohlerman NJ (1985). Magnocellular red nucleus activity during different types of limb movement in the macaque monkey. J Physiol.

[CR40] Gibson AR, Horn KM, Stein JF, Van Kan PL (1996). Activity of interpositus neurons during a visually guided reach. Can J Physiol Pharmacol.

[CR41] Giolli RA, Gregory KM, Suzuki DA, Blanks RH, Lui F, Betelak KF (2001). Cortical and subcortical afferents to the nucleus reticularis tegmenti pontis and basal pontine nuclei in the macaque monkey. Vis Neurosci.

[CR42] Glickstein M, May JG, Mercier BE (1985). Corticopontine projection in the macaque: the distribution of labelled cortical cells after large injections of horseradish peroxidase in the pontine nuclei. J Comp Neurol.

[CR43] Gregg TR, Siegel A (2003). Differential effects of NK1 receptors in the midbrain periaqueductal gray upon defensive rage and predatory attack in the cat. Brain Res.

[CR44] Grillner S, Hongo T (1972). Vestibulospinal effects on motoneurones and interneurones in the lumbosacral cord. Prog Brain Res.

[CR45] Guo W, Fan S, Xiao D (2021). A Brainstem reticulotegmental neural ensemble drives acoustic startle reflexes. Nat Commun.

[CR46] Hariz M, Blomstedt P (2022). Deep brain stimulation for Parkinson's disease. J Intern Med.

[CR47] Hazrati LN, Parent A (1992). Projection from the deep cerebellar nuclei to the pedunculopontine nucleus in the squirrel monkey. Brain Res.

[CR48] Hess BJ, Blanks RH, Lannou J, Precht W (1989). Effects of kainic acid lesions of the nucleus reticularis tegmenti pontis on fast and slow phases of vestibulo-ocular and optokinetic reflexes in the pigmented rat. Exp Brain Res.

[CR49] Holstege G, Blok BF, Ralston DD (1988). Anatomical evidence for red nucleus projections to motoneuronal cell groups in the spinal cord of the monkey. Neurosci Lett.

[CR50] Hongo T, Kudo N, Tanaka R (1975). The vestibulospinal tract: crossed and uncrossed effects on hindlimb motoneurones in the cat. Exp Brain Res.

[CR51] Ilinsky IA, Kultas-Ilinsky K (1984). An autoradiographic study of topographical relationships between pallidal and cerebellar projections to the cat thalamus. Exp Brain Res.

[CR52] Inglis WL, Winn P (1995). The pedunculopontine tegmental nucleus: where the striatum meets the reticular formation. Prog Neurobiol.

[CR53] Jaarsma D, Ruigrok TJ, Caffe R, Cozzari C, Levey AI, Mugnaini E, Voogd J (1997). Cholinergic innervation and receptors in the cerebellum. Prog Brain Res.

[CR54] Jackman SL, Chen CH, Offermann HL (2020). Cerebellar Purkinje cell activity modulates aggressive behavior. Elife.

[CR55] Kaneko CR, Fuchs AF (2006). Effect of pharmacological inactivation of nucleus reticularis tegmenti pontis on saccadic eye movements in the monkey. J Neurophysiol.

[CR56] Kawamura K, Hashikawa T (1981). Projections from the pontine nuclei proper and reticular tegmental nucleus onto the cerebellar cortex in the cat. An Autoradiographic Study. J Comp Neurol.

[CR57] Lanciego JL, Luquin N, Obeso JA (2012). Functional neuroanatomy of the basal ganglia. Cold Spring Harb Perspect Med.

[CR58] Lawrence DG, Kuypers HG (1968). The functional organization of the motor system in the monkey. I. The effects of bilateral pyramidal lesions. Brain.

[CR59] Maeda M, Maunz RA, Wilson VJ (1975). Input from the labyrinth to cat forelimb motoneurones. J Physiol.

[CR60] Marsden CD, Obeso JA (1994). The functions of the basal ganglia and the paradox of stereotaxic surgery in Parkinson's disease. Brain.

[CR61] Matsunami K (1987). Neuronal activity in nuclei pontis and reticularis tegmenti pontis related to forelimb movements of the monkey. Neurosci Res.

[CR62] Matsuyama K, Drew T (1997). Organization of the projections from the pericruciate cortex to the pontomedullary brainstem of the cat: a study using the anterograde tracer Phaseolus vulgaris-leucoagglutinin. J Comp Neurol.

[CR63] Matsuyama K, Jankowska E (2004). Coupling between feline cerebellum (fastigial neurons) and motoneurons innervating hindlimb muscles. J Neurophysiol.

[CR64] McBride RL, Larsen KD (1980). Projections of the feline globus pallidus. Brain Res.

[CR65] McCall AA, Miller DM, Yates BJ (2017). Descending influences on vestibulospinal and vestibulosympathetic reflexes. Front Neurol.

[CR66] McCurdy ML, Hansma DI, Houk JC, Gibson AR (1987). Selective projections from the cat red nucleus to digit motor neurons. J Comp Neurol.

[CR67] McElvain LE, Chen Y, Moore JD (2021). Specific populations of basal ganglia output neurons target distinct brain stem areas while collateralizing throughout the diencephalon. Neuron.

[CR68] Mihailoff GA, Kosinski RJ, Azizi SA, Border BG (1989). Survey of noncortical afferent projections to the basilar pontine nuclei: a retrograde tracing study in the rat. J Comp Neurol.

[CR69] Miller RA, Strominger NL (1977). An experimental study of the efferent connections of the superior cerebellar peduncle in the rhesus monkey. Brain Res.

[CR70] Mori S, Matsui T, Kuze B, Asanome M, Nakajima K, Matsuyama K (1999). Stimulation of a restricted region in the midline cerebellar white matter evokes coordinated quadrupedal locomotion in the decerebrate cat. J Neurophysiol.

[CR71] Mori S, Matsui T, Mori F, Nakajima K, Matsuyama K (2000). Instigation and control of treadmill locomotion in high decerebrate cats by stimulation of the hook bundle of Russell in the cerebellum. Can J Physiol Pharmacol.

[CR72] Mori F, Okada KI, Nomura T, Kobayashi Y (2016). The pedunculopontine tegmental nucleus as a motor and cognitive interface between the cerebellum and basal ganglia. Front Neuroanat.

[CR73] Murray AJ, Croce K, Belton T, Akay T, Jessell TM (2018). Balance control mediated by vestibular circuits directing limb extension or antagonist muscle co-activation. Cell Rep.

[CR74] Nauta HJ (1979). Projections of the pallidal complex: an autoradiographic study in the cat. Neuroscience.

[CR75] Nauta HJ, Cole M (1978). Efferent projections of the subthalamic nucleus: an autoradiographic study in monkey and cat. J Comp Neurol.

[CR76] Nieoullon A, Gahery Y (1978). Influence of pyramidotomy on limb flexion movements induced by cortical stimulation and on associated postural adjustment in the cat. Brain Res.

[CR77] Orlovsky GN (1972). Activity of vestibulospinal neurons during locomotion. Brain Res.

[CR78] Ossowska K (2020). Zona incerta as a therapeutic target in Parkinson's disease. J Neurol.

[CR79] Plaha P, Ben-Shlomo Y, Patel NK, Gill SS (2006). Stimulation of the caudal zona incerta is superior to stimulation of the subthalamic nucleus in improving contralateral parkinsonism. Brain.

[CR80] Pogossian VI, Fanardjian VV (1992). Organization of afferent projections to the ventral and dorsal regions of the cat lateral vestibular nucleus: an HRP study. J Vestib Res.

[CR81] Pong M, Horn KM, Gibson AR (2002). Spinal projections of the cat parvicellular red nucleus. J Neurophysiol.

[CR82] Pretegiani E, Optican LM (2017). Eye movements in Parkinson's disease and inherited parkinsonian syndromes. Front Neurol.

[CR83] Rahimpour S, Gaztanaga W, Yadav AP (2021). Freezing of gait in Parkinson's disease: invasive and noninvasive neuromodulation. Neuromodulation.

[CR84] Robinson FR, Houk JC, Gibson AR (1987). Limb specific connections of the cat magnocellular red nucleus. J Comp Neurol.

[CR85] Romano V, Reddington AL, Cazzanelli S (2020). Functional convergence of autonomic and sensorimotor processing in the lateral cerebellum. Cell Rep.

[CR86] Ruggiero D, Batton RR, Jayaraman A, Carpenter MB (1977). Brain stem afferents to the fastigial nucleus in the cat demonstrated by transport of horseradish peroxidase. J Comp Neurol.

[CR87] Sakai ST, Inase M, Tanji J (1996). Comparison of cerebellothalamic and pallidothalamic projections in the monkey (Macaca fuscata): a double anterograde labeling study. J Comp Neurol.

[CR88] Sastre JP, Sakai K, Jouvet M (1981). Are the gigantocellular tegmental field neurons responsible for paradoxical sleep?. Brain Res.

[CR89] Serapide MF, Parenti R, Panto MR, Zappala A, Cicirata F (2002). Multiple zonal projections of the nucleus reticularis tegmenti pontis to the cerebellar cortex of the rat. Eur J Neurosci.

[CR90] Shaikh MB, Barrett JA, Siegel A (1987). The pathways mediating affective defense and quiet biting attack behavior from the midbrain central gray of the cat: an autoradiographic study. Brain Res.

[CR91] Sheard MH, Flynn JP (1967). Facilitation of attack behavior by stimulation of the midbrain of cats. Brain Res.

[CR92] Shinoda Y, Ohgaki T, Futami T (1986). The morphology of single lateral vestibulospinal tract axons in the lower cervical spinal cord of the cat. J Comp Neurol.

[CR93] Shinoda Y, Sugiuchi Y, Futami T, Kakei S, Izawa Y, Na J (1996). Four convergent patterns of input from the six semicircular canals to motoneurons of different neck muscles in the upper cervical cord. Ann N Y Acad Sci.

[CR94] Smith Y, Hazrati LN, Parent A (1990). Efferent projections of the subthalamic nucleus in the squirrel monkey as studied by the PHA-L anterograde tracing method. J Comp Neurol.

[CR95] Snider RS, Eldred E (1952). Cerebrocerebellar relationships in the monkey. J Neurophysiol.

[CR96] Stenmark Persson R, Nordin T, Hariz GM, Wardell K, Forsgren L, Hariz M, Blomstedt P (2022). Deep brain stimulation of caudal zona incerta for Parkinson's disease: one-year follow-up and electric field simulations. Neuromodulation.

[CR97] Suzuki DA, Yamada T, Hoedema R, Yee RD (1999). Smooth-pursuit eye-movement deficits with chemical lesions in macaque nucleus reticularis tegmenti pontis. J Neurophysiol.

[CR98] Takakusaki K, Chiba R, Nozu T, Okumura T (2016). Brainstem control of locomotion and muscle tone with special reference to the role of the mesopontine tegmentum and medullary reticulospinal systems. J Neural Transm (vienna).

[CR99] Tovote P, Esposito MS, Botta P (2016). Midbrain circuits for defensive behaviour. Nature.

[CR100] Udo M, Matsukawa K, Kamei H (1979). Effects of partial cooling of cerebellar cortex at lobules V and IV of the intermediate part in the decerebrate walking cats under monitoring vertical floor reaction forces. Brain Res.

[CR101] van Donkelaar P, Stein JF, Passingham RE, Miall RC (2000). Temporary inactivation in the primate motor thalamus during visually triggered and internally generated limb movements. J Neurophysiol.

[CR102] van Kan PL, Horn KM, Gibson AR (1994). The importance of hand use to discharge of interpositus neurones of the monkey. J Physiol.

[CR103] Voogd J (2016). Deiters' nucleus. its role in cerebellar ideogenesis: the ferdinando rossi memorial lecture. Cerebellum.

[CR104] Wall PD (1970). The sensory and motor role of impulses travelling in the dorsal columns towards cerebral cortex. Brain.

[CR105] Wall PD, Noordenbos W (1977). Sensory functions which remain in man after complete transection of dorsal columns. Brain.

[CR106] Wurtz RH, Hikosaka O (1986). Role of the basal ganglia in the initiation of saccadic eye movements. Prog Brain Res.

[CR107] Yamada T, Suzuki DA, Yee RD (1996). Smooth pursuitlike eye movements evoked by microstimulation in macaque nucleus reticularis tegmenti pontis. J Neurophysiol.

[CR108] Zangger P, Schultz W (1978). The activity of cells of nucleus reticularis tegmenti pontis during spontaneous locomotion in the decorticate cat. Neurosci Lett.

[CR109] Zhang SP, Bandler R, Carrive P (1990). Flight and immobility evoked by excitatory amino acid microinjection within distinct parts of the subtentorial midbrain periaqueductal gray of the cat. Brain Res.

